# The Balance of Expression of Dihydroflavonol 4-reductase and Flavonol Synthase Regulates Flavonoid Biosynthesis and Red Foliage Coloration in Crabapples

**DOI:** 10.1038/srep12228

**Published:** 2015-07-20

**Authors:** Ji Tian, Zhen-yun Han, Jie Zhang, YuJing Hu, Tingting Song, Yuncong Yao

**Affiliations:** 1 Department of Plant Science and Technology, Beijing University of Agriculture, Beijing, China; 2 Key Laboratory of New Technology in Agricultural Application of Beijing, Beijing University of Agriculture, Beijing, China

## Abstract

Red leaf color is an attractive trait of *Malus* families, including crabapple (*Malus* spp.); however, little is known about the molecular mechanisms that regulate the coloration. Dihydroflavonols are intermediates in the production of both colored anthocyanins and colorless flavonols, and this current study focused on the gene expression balance involved in the relative accumulation of these compounds in crabapple leaves. Levels of anthocyanins and the transcript abundances of the anthocyanin biosynthetic gene, dihydroflavonol 4-reductase (*McDFR*) and the flavonol biosynthetic gene, flavonol synthase (*McFLS*), were assessed during the leaf development in two crabapple cultivars, ‘Royalty’ and ‘Flame’. The concentrations of anthocyanins and flavonols correlated with leaf color and we propose that the expression of *McDFR* and *McFLS* influences their accumulation. Further studies showed that overexpression of *McDFR*, or silencing of *McFLS,* increased anthocyanin production, resulting in red-leaf and red fruit peel phenotypes. Conversely, elevated flavonol production and green phenotypes in crabapple leaves and apple peel were observed when *McFLS* was overexpressed or *McDFR* was silenced. These results suggest that the relative activities of McDFR and McFLS are important determinants of the red color of crabapple leaves, via the regulation of the metabolic fate of substrates that these enzymes have in common.

Flavonoids are a class of plant secondary metabolites that collectively have diverse functions, including providing protection against abiotic stresses, particularly UV-irradiation, and biotic factors, such as phytophagous insects, as well as attracting pollinators^1^,^2^. They also have considerable value as components of the human diet^3^ and are used in the pharmaceutical industry since they have antioxidative, anticancer and anti-inflammatory properties^4^,^5^. Flavonoids have been extensively studied in a range of land plants and have been divided into nine structural subclasses: chalcones, flavones, flavonols, dihydroflavonols, flavandiols, anthocyanins, proanthocyanidins, flavonoid isoflavonoids and aurones^5^. Moreover, many genes involved in the flavonoid biosynthetic pathway have been identified and characterized. For example, the genes encoding chalcone synthase (CHS), chalcone isomerase (CHI), F3H flavanone 3-hydroxylase (F3H), flavonoid 3′-monooxygenase (F3′H), dihydroflavonol 4-reductase (DFR), anthocyanidin synthase (ANS), uridine diphosphate glucose-flavonoid 3-O-glucosyl transferase (UFGT), flavonol synthase (FLS), leucoanthocyanidin reductase (LAR) and anthocyanidin reductase (ANR) have been characterized in several plant species^6^,^7^,^8^,^9^,^10^,^11^,^12^,^13^,^14^.

The flavonoid biosynthetic pathway lies downstream of the phenylpropanoid pathway and leads to the formation of anthocyanins and flavonols (Fig. 1). Chalcone synthase (CHS; EC 2.3.1.74) uses beta-coumaroyl-CoA and 3 Malonyl-CoA as substrates to form naringenin chalcone^15^. This condensation reaction is a key step in the pathway leading to the formation of flavonoids^16^. Next, the F3H converts naringenin to dihydrokaempferol. Then dihydrokaempferol and dihydroquercetin are converted to kaempferol and quercetin by FLS, respectively. DFR and FLS also catalyze competing reactions to generate products leading to the spectrum of downstream anthocyanins and flavonols^17^,^18^.

MYB family transcriptional factors are known to be involved in regulating the expression expression of flavonoid biosynthesis genes^19^. For example, in *Arabidopsis thaliana*, AtMYBL2 interacts with TT8 (TRANSPARENT TESTA 8) to reduce anthocyanin biosynthesis^20^. In the context of flavonoid biosynthesis, AtMYB12 is thought to effect flavonol production^19^,^21^ by regulation *CHS*, *CHI*, *F3H* and *FLS1* gene expression levels^22^. In addition, the expression of three allelic apple (*Malus* × *domestic*) genes (*MYB10, MYB1* and *MYBA*) was shown to strongly correlate with the accumulation of anthocyanins in fruit^23^,^24^,^25^.

The pigmentation of many plant organs results from the presence of some of these flavonoid classes and, in many instances, a primary determinant of color is the accumulation of anthocyanins. The synthesis of anthocyanins occurs via a key branch in the flavonoid biosynthesis pathway, involving the action of the enzyme DFR on the dihydroflavonols dihydroquercetin, dihydrokaempferol or dihydromyricetin. Indeed, *DFR* genes have been shown to play an important role in determining the total anthocyanin content in *A. thaliana*^26^,^27^. Additionally, the expression level of the *DFR* gene *TfDFR1* has been shown to be positively correlated with red pigment accumulation in the petals of tulip (*Tulipa fosteriana*)^28^. It has also been reported that the expression level of *DFR* positively correlates with the abundance of anthocyanins in peanut (*Arachis hypogaea*)^29^.

Flavonols provide another important co-pigment in the colorful organs of terrestrial plants, such as the yellow petals of *Lathyrus chrysanthus*^30^, and they also influence pollen tube growth^31^. Flavonols are derived from 2, 3-dihydroquercetin and their formation is catalyzed by FLS, which belongs to the 2-oxoglutarate dependent dioxygenase family^4^,^32^. Following the identification of an *FLS* gene from *Petunia hybrida*^33^, homologous *FLS* genes have been identified from *A. thaliana*, *Solanum tuberosum*, *Matthiola incana*, *Malus domestica* and *Eustoma russellianum.* In addition, it has been reported that increases in transcript levels of an *FLS* gene from satsuma mandarin (*Citrus unshiu* Marc.), *CitFLS*, in the fruit peel correlate with flavonol accumulation during fruit development^34^. Recently, it has been found that major floral color changes are a consequence of *FLS* expression in petunia (*Petunia hybrida Vilm*.), *Lisianthus* (*Eustoma grandiflorum*) and camellia (*C. nitidissima*)^33^,^35^,^36^,^37^. In contrast, an indirect effect of a camellia *FLS* gene (*CnFLS1*) on anthocyanin accumulation during floral coloration was suggested following an experiment where its overexpression in transgenic tobacco (*N. benthamiana*) plants resulted in an increase in flavonol content, but a reduction in anthocyanin levels in petals^37^.

Leaf color is a key determinant of the commercial value of many ornamental plant species; however, much remains to be learnt about the mechanisms of color formation in leaves at the molecular level. The study of pigmentation mechanisms in leaves is therefore significant for both breeding and genetic engineering of ornamental plants. An example of an important ornamental woody plant is *Malus* crabapple, which belongs to the Rosaceae, *Malus Mill* family. The numerous plant landscape species in this family provide an excellent source of research material for studying the mechanism of color formation and accumulation, due to their colorful leaves, flowers and fruits^38^. To date, little is known about the mechanism of anthocyanin and flavonol biosynthesis in ornamental crabapples.

In this current study, we investigated the function of the crabapple DFR (*McDFR*) and FLS (*McFLS*) genes in regulating leaf color in different cultivars. We overexpressed and silenced each gene to determine their interaction in controlling flavonol and anthocyanin biosynthesis, and evaluated the gene expression ratio of *McDFR* and *McFLS* that is required for leaf color production. We also discuss the metabolic flux between McDFR and McFLS during flavonoid biosynthesis in leaves and fruit. We propose that the finding from this study will assist future attempts to enhance anthocyanin or flavonol accumulation in the leaves of target ornamental species by altering the balance between the McDFR and McFLS enzyme activities.

## Results

### The anthocyanin and flavonol content of the leaves of two crabapple cultivars

Two extreme leaf color cultivars, ‘Royalty’ and ‘Flame’, have ever-red and ever-green leaves, respectively. We evaluated the abundance of anthocyanins and flavonols in the leaves of these cultivars at 5 development stages of the crabapple leaf growing season by high-performance liquid chromatography (HPLC) (Fig. 2). The chromatography results showed that cyanidin 3-O-glucoside was the predominant anthocyanin, and we found that that the major flavonols were quercetin derived compounds, such as quercetin 3-O-diglucoside, quercetin 3-O-glucoside and quercetin 3-O-glycosidase isomer (Fig. 2B). As shown in Fig. 2C, anthocyanin levels in the ever-red leaves of ‘Royalty’ were significantly higher than those in the ever-green leaves of ‘Flame’. A gradual decrease in anthocyanin content was observed in ‘Royalty’ leaves during their development, while anthocyanins were almost undetectable in ‘Flame’ leaves. In contrast, the abundance of flavonols increased during the development of ‘Flame’ leaves, except at stage 5.

### The expression levels of anthocyanin and flavonol biosynthetic genes in the leaves of the two crabapple cultivars

To gain insight into the relationship between the expression patterns of anthocyanin biosynthetic genes and anthocyanin/flavonol accumulation, the transcript levels of the key anthocyanin biosynthetic gene *McDFR*, and the flavonol biosynthesis gene *McFLS,* were determined by qRT-PCR. The expression of *McDFR* decreased during the development of both ever-red and ever-green leaves (Fig. 3). In contrast, the transcript levels of *McFLS* showed an increase in the early developmental stages, and then decreased in stages 4 and 5 in ever-red leaves (Fig. 2A), while only a slight decrease was seen in the last stage of the ever-green leaves (Fig. 3B). This suggested that *McDFR* expression positively correlated with anthocyanin accumulation during the development of ever-red leaves, and that *McFLS* expression and flavonol accumulation were positively correlated in leaves of both cultivars.

### McDFR plays an important role in the anthocyanin biosynthetic pathway

To further investigate the function of *McDFR*, we suppressed its expression in the leaves of the red-leafed crabapple cultivar ‘Royalty’ by virus-induced gene silencing (VIGS) using the TRV vector^39^. Leaves infected with the virus containing the TRV-*McDFR* construct began to exhibit a green color at 14 days post-infection (dpi), while a more severe phenotype was detected in the new buds of TRV-*McDFR* infected stem tips at 35 dpi (Fig. 4A). We next analyzed the variation in flavonoid content of the infected leaves by HPLC and observed that in the *McDFR* silenced areas of the leaves; the levels of anthocyanin were much lower than in non-silenced leaves, while the flavonol content showed a significant increase (Fig. 4B). We also confirmed, by qRT-PCR analysis, that the abundance of endogenous *McDFR* transcripts was greatly reduced in TRV-*McDFR* infected leaves. The expression levels of other flavonoid biosynthetic genes, namely *CHS* (*McCHS*) and *F3′H* (*McF3′H*) were similar in the control and infected leaves, while silencing of the *McDFR* gene increased the transcript levels of phenylalanine ammonia lyase (*PAL*; *McPAL*), *CHI* (*McCHI*), *FSH* (*McF3H*), *FLS* (*McFLS*), *ANS* (*McANS*) and *UFGT* (*McUFGT*) genes (Fig. 4C).

We also transiently over-expressed the *McDFR* gene in the stem tips of the ever-green cultivar ‘Strawberry Jelly’, which promoted anthocyanin accumulation at 20 dpi, and a deep green coloration in most of the new buds (Fig. 4A). The anthocyanin content showed a slight increase in the *McDFR*-overexpressing plants (Fig. 4B) and we confirmed that these plants indeed had higher *McDFR* transcript levels in the new buds (Fig. 4C). We also detected an increase in the transcript levels of anthocyanin biosynthetic genes and a decrease in *McFLS* expression, compared with the non-transformed plants (Fig. 4C).

Collectively, these results indicated that *McDFR* expression is associated with red color formation in crabapple leaves, and that changes in *McDFR* expression can affect the expression of downstream genes (e.g. *ANS* and *UFGT*) involved in the anthocyanin biosynthetic pathway.

### McFLS is involved in flavonol biosynthesis in crabapple leaves

To confirm the prediction, based on sequence homology, that *McFLS* is a key flavonol biosynthetic gene, we suppressed its expression in the leaves of ‘Strawberry Jelly’ using the VIGS system and the TRV vector. Approximately 14 days after *Agrobacterium* infiltration, red coloration was seen in the margin and other areas of the infected leaves (Fig. 5A). HPLC analysis confirmed that the levels of anthocyanins were significantly higher in the silenced leaves than in control leaves infiltrated with TRV alone (Fig. 5B). Finally, as seen in Fig. 4C, the expression of *McPAL*, *McDFR* and *McANS* was up-regulated in infected leaves.

We also transiently overexpressed *McFLS* in the stem tips of the ever-red leaves of ‘Royalty’. Crabapple stem tips transformed with *35S::McFLS* developed new buds with a green color (Fig. 5A), indicating that anthocyanin synthesis was suppressed. This was confirmed by HPLC analysis, which also revealed an increase in flavonol content (Fig. 5B). As expected, we observed an increase in *McFLS* transcript levels in green buds infiltrated with *35S::McFLS* compared to control leaves. This elevated expression of *McFLS* caused an approximately 1.6-fold decrease in *McDFR* transcript abundance, and we observed lower levels of expression of other anthocyanin biosynthetic genes (Fig. 5C). To summarize, these results suggested that *McFLS* expression can promote flavonol accumulation by inhibiting the expression of *McDFR*, and that *McFLS* has a competitive relationship with *McDFR* in the flavonol biosynthetic pathway.

### Variation in the expression of *McDFR* and *McFLS* in apple fruit

To further characterize the roles of *McDFR* and *McFLS*, we infected apple (*Malus* × *domestica* ‘Fuji’) fruits with transgenic *Agrobacterium* harboring TRV-*McDFR*, TRV-*McFLS*, pBI121-*McDFR* or pBI121-*McFLS* constructs (Figs 6 and 7). A large increase in anthocyanin accumulation was observed at the sites of TRV-*McFLS* and pBI121-*McDFR* over-expression, while the areas of TRV-*McDFR* and pBI121-*McFLS* over-expression showed a yellow or white coloration (Figs 6A and 7A). HPLC quantification of the anthocyanin and flavonol content of the infected areas confirmed that the variation in flavonoid content correlated with the observed variation in fruit color (Figs 6B and 7B). Transcript expression analysis of the transiently expressing tissues further revealed that the up-regulation of *McDFR* expression, or the down-regulation of *McFLS* expression, was accompanied by a proportional increase in the expression levels of *McDFR* as well as some of the genes involved in anthocyanin biosynthesis (*McCHS*, *McCHI*, *McF3H*, *McF3′H*, *McDFR*, *McANS* and *McUFGT*), and a decrease in the expression levels of *McFLS*. Importantly, up-regulation of *McFLS* expression or down-regulation of *McDFR* expression resulted in fading leaf color and decrease *McDFR* gene expression (Figs 6C and 7C). Finally, we compared the relationship between the expression of *McDFR* and the content of anthocyanins, and the results were consistent with the red color formation in leaves and fruit; as well as between the levels of *McFLS* and the content of flavonols (Figs 5 and 6).

## Discussion

Due to the central role that the DFR enzyme plays in the anthocyanin biosynthetic pathway, *DFR* genes have been studied in several monocotyledonous and dicotyledonous species, such as *Forsythia intermedia*^40^, *Torenia fournieri*^31^, *Triticum aestivum*^41^, *Vitis vinifera*^42^ and *Ascocenda* spp.^43^, and in some cases at the transcriptional level^44^. The key structural gene, *FLS,* completes the last step of flavonol synthesis. To our knowledge, although *FLS* and *DFR* have been identified and characterized in many land plant species, these genes have yet to be studied in ornamental crabapples. There is growing evidence that anthocyanins and flavonols to contribute to the ornamental and economic value of crabapples, which have highly colorful leaves, fruits and flowers^38^. In this study we explored the expression and function of *McDFR* and *McFLS* in the biosynthesis and accumulation of anthocyanins and flavonols.

### *McDFR* and *McFLS* expression are coordinated and correlate with the accumulation of anthocyanins and flavonols

In this study, the content of anthocyanins in the leaves of two crabapple cultivars was evaluated, and we observed that it increased in parallel with red pigmentation in several organs/tissues. Furthermore, the anthocyanin levels showed a positive correlation with the expression of *McDFR*, but a negatively correlation with the expression of *McFLS* (Table 1). This result is congruent with a recent study showing a negative correlation of *CnFLS1* expression and anthocyanin synthesis during floral coloration in the petals of transgenic tobacco expressing this gene^37^. We infer from these observations that there is a competitive relationship between *McDFR* and *McFLS* in flavonoid biosynthesis.

### The function of *McDFR* and *McFLS* in flavonoid biosynthesis

Genetic transformation has been used to test the functions of several genes in the flavonoid biosynthetic pathway in model experimental plants, such as tobacco and *A. thaliana*, as well as in some crop species. For example, overexpression of petunia *CHI* in tomato fruit was reported to lead to an ~65% increase in flavonol levels^45^, while silencing of a *CHS* gene in apple fruit resulted in changes in growth and developmental phenotypes^46^. In *Gentiana triflora* and apples (*Malus* spp.), the silencing of an *ANS* gene caused a reduction in anthocyanin content and, consequently, a much weaker color^11^. However, few studies to date have focused on genetic transformation using *DFR* or *FLS* genes and none has targeted woody ornamental species, such as crabapple.

Virus-induced gene silencing (VIGS) is a technology that allows the analysis of genes function by suppressing the expression of target genes. In a previous study, we assessed the effect of gene silencing in several plant species, including *Nicotiana benthamiana*, rose (*Rose hybrida*) and strawberry (*Fragaria* × *ananassa*)^47^,^48^,^49^. Here, we used tissue cultured seedling buds from different crabapple cultivars (‘Royalty’, ‘Strawberry Jelly’) to assess the function of *McDFR* and *McFLS* through VIGS and overexpression approaches. We determined that silencing of *McFLS* or overexpression of *McDFR* promoted the accumulation of anthocyanins, while the opposite results were obtained when *McFLS* was overexpressed or *McDFR* expression was silenced. In addition, the abundance of flavonols increased when *McFLS* was overexpressed or *McDFR* was silenced. However, it is interesting that levels of flavonols were slightly elevated in transgenic crabapple leaves that were transiently overexpressing *McDFR* or in which *McFLS* expression was silenced. This phenomenon may be explained if *McFLS* was still expressed but at lower levels degree in *McDFR* overexpressed or *McFLS* silenced crabapple plants. In this scenario the lower expression level of *McFLS* in these transgenic crabapple plants may have resulted in reduced accumulation flavonol, but not a complete block in flavonol biosynthesis. Moreover, correlation coefficients indicated that the levels of expression of *McDFR* negatively correlated with those of *McFLS*, as well as with the abundance of anthocyanins and flavonols (Table 1). We propose that there is a competitive relationship between the expression *McDFR* and *McFLS* that results in the production of different classes of flavonoid compounds (i.e. anthocyanins or flavonols) (Figs 4, 5, 6, 7).

Since dihydroflavonols are substrates for DFR and FLS, they lie at an important branch point in flavonoid biosynthesis, where precursor substrates are channeled toward either anthocyanin or flavonol production. In this regard, the regulation of *DFR* expression and the competing DFR and FLS activities may be particularly important. We conclude that the expression of *McDFR* and *McFLS* may represent a key mechanism for regulating color in crabapple leaves.

### A possible explanation for the expression patterns of *McDFR* and *McFLS* in two contrasting varieties

R2R3-MYB transcription factors (TFs), which belong to one of the largest plant TF families, are known to be involved in regulating the biosynthesis of anthocyanins and flavonols in *A. thaliana*^50^. A previously study reported that over-expression of the *A. thaliana* TF AtMYB12 in tobacco (*Nicotiana tabacum*) resulted in higher levels of flavonols and increased expression of the *FLS* gene^51^. Moreover, when AtMYB12 was over-expressed in a tissue-specific manner in tomato, the flavonol biosynthesis pathway was activated^51^. However, expression of the *DFR* gene was not induced in *AtMYB12* and *AtMYB111* over-expressing transgenic plants, as well as in *AtMYB111* transgenic lines^1^,^44^,^51^. In apple and crabapple, MYB10, which regulates anthocyanin accumulation and coloration of various organ (e.g. fruit, petals and leaves), can activate the expression of *DFR* and bind to several the promoters of several anthocyanin biosynthetic genes^52^,^53^. In grape berries, expression of the regulatory gene *VvMYBF1* is light inducible, and is involved in the control of *VvFLS1* transcription and flavonol synthesis in fruit^54^. Thus, we speculated that MYB TFs may similarly regulate flavonoid biosynthesis in crabapple, and that their expression levels may vary at different development stages or in response to different environment conditions. Moreover, changes in the transcript levels of MYB TFs control the biosynthesis of flavonoids, by regulating the expression of various members of the flavonoid biosynthetic pathway. We propose that MYB TFs promote anthocyanin biosynthesis by increasing the transcript levels of *DFR* during fruit or leaf development, leading to red coloration. However, in response to environmental stresses, MYB TFs activate the transcription of *FLS* resulting in increased production of flavonols.

## Conclusion

In this study, we investigated the functions of *McDFR* and *McFLS* in regulating leaf color in different crabapple cultivars. We demonstrated that the competitive relationship between the expression of *McDFR* and *McFLS* is important for anthocyanin and flavonol synthesis. In addition, the expression of *McDFR* and *McFLS* correlated with anthocyanin and flavonol accumulation, as well as with leaf color. The work described in this report may suggest strategies to genetically modify ornamental plants in order to enhance or modulate flavonoid accumulation.

## Methods

### Plant material and growth conditions

The plant material used included three *Malus* crabapple cultivars: (1) *Malus* cv. ‘Royalty’, an ever-red leaf cultivar; (2) *Malus* cv. ‘Flame’, an ever-green leaf cultivar. Five year old trees of these cultivars grafted on *M. hupehensis* were planted in the Crabapple Germplasm Resources Nursery in Beijing University of Agriculture; and (3) *Malus* cv. ‘Strawberry Jelly’, an ever-green leaf cultivar, the explants of which were harvested from one-year old branches before spring bud germination, cultured on Murashige and Skoog medium supplemented with 0.1 mg/L 6-Benzylaminopurine (6-BA) and 2 mg/L (2,4-dichlorophenoxy) acetic acid (2,4-D) at 23 °C with a 16 h light (200 μmol s^–1^ m^–2^) /8 h dark period. Plants were grown in a greenhouse at 27 °C under constant illumination.

**Figure 1:**
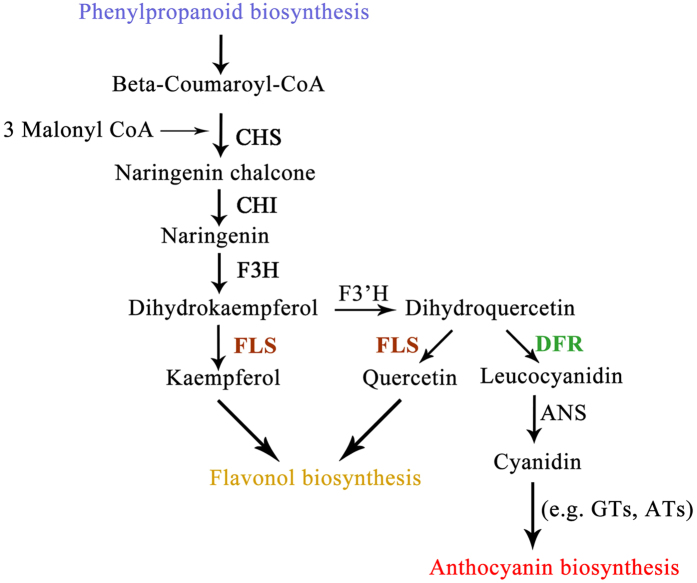
Diagrammatic representation of the flavonoid biosynthetic pathway in plant. Enzymes required for flavonol and anthocyanin synthesis: CHS, chalcone synthase; CHI, chalcone isomerase; F3H, flavanone 3-hydroxylase; FLS, flavonol synthase; DFR, dihydroflavonol 4-reductase; F’3H, flavonoid 3′-monooxygenase; ANS, anthocyanidin synthase; GTs, glucosyltransferases; ATs, anthocyanin aromatic acyltransferases.

**Figure 2:**
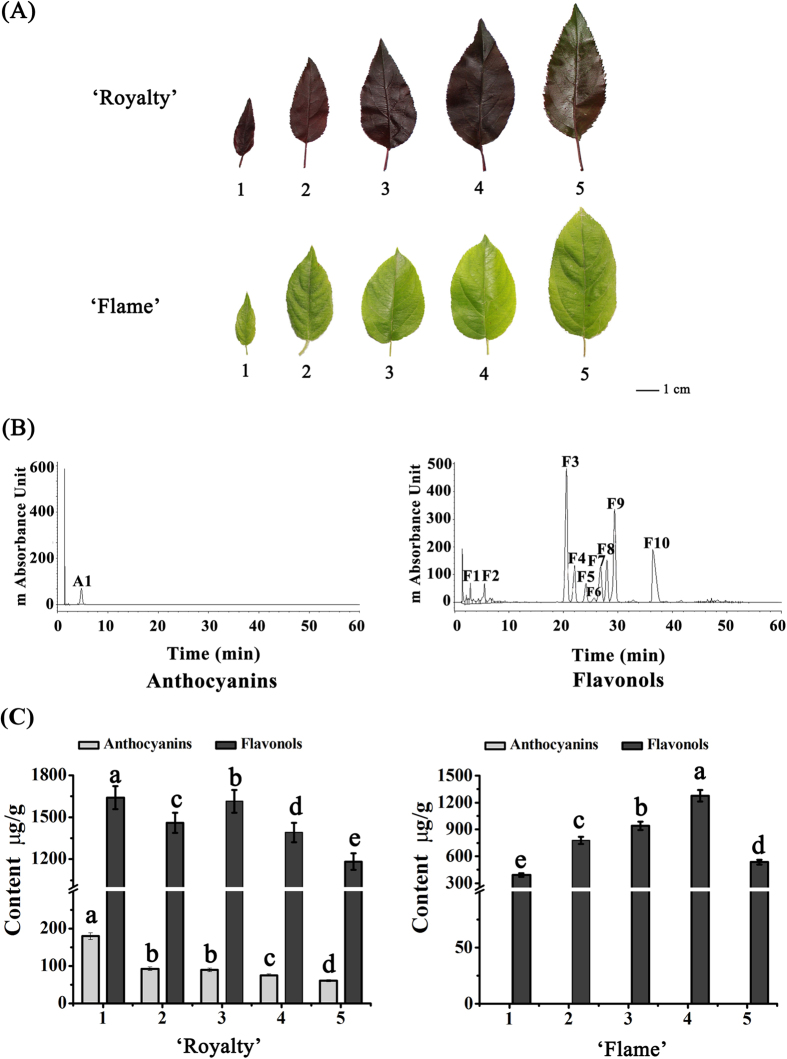
Analysis of flavonoid accumulation in 5 developmental stages of leaves from the *Malus* crabapple cultivars ‘Royalty’ and ‘Flame’. S1, 3 days after budding; S2, 9 days after budding; S3, 15 days after budding; S4, 21 days after budding; S5, 30 days after budding. (**A**) Five leaf developmental stages used for the analysis. (**B**) HPLC analysis of methanol extracts from crabapple leaf. A1, cyanidin 3-O-glucoside; F1, quercetin 3-O-diglucoside; F2, procyanidin dimer; F3, quercetin 3-O-glucoside; F4, quercetin 3-O-glycosidase isomer; F5, avicularin; F6, acetyl quercetin 3-O-glucoside; F7, acetyl quercetin 3-O-glycosidase isomer; F8, avicularin isomer; F9, quercetin 3-O-rhamnoside; F10, phloridzin. (**C**) The total anthocyanins content and total flavonols content in 5 developmental stages of leaves of the two crabapple cultivars. Error bars indicate the standard error of the mean ± SE of three replicate measurements. Different letters above the bars indicate significantly different values (*P* < 0.05) calculated using one-way analysis of variance (ANOVA) followed by a Duncan’s multiple range test.

**Figure 3:**
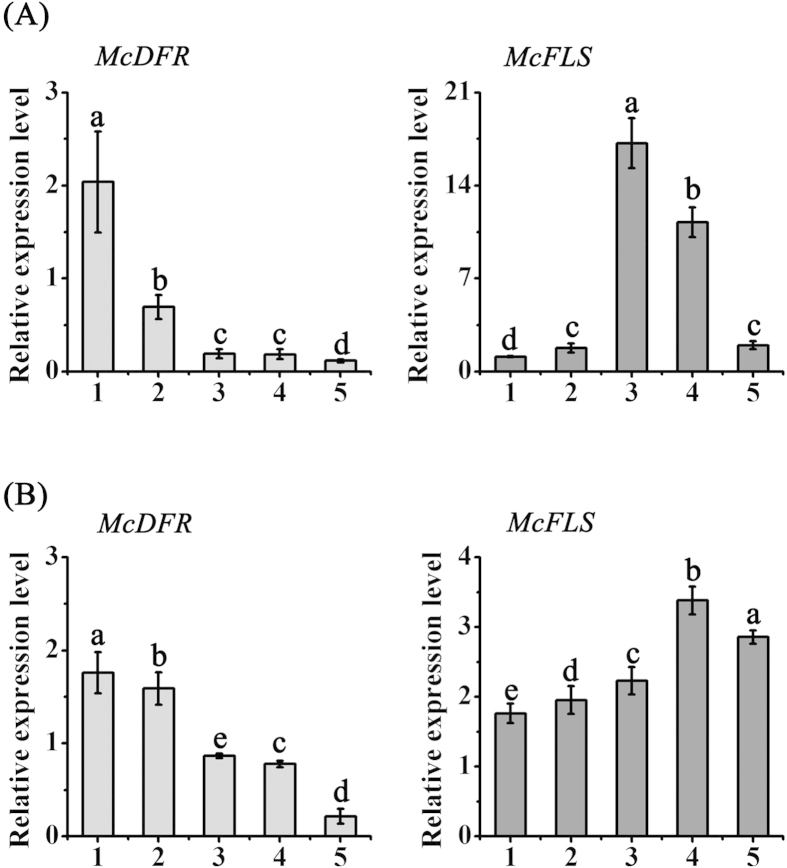
The relative expression level of *McDFR* and *McFLS* transcripts at different development stages of ‘Royalty’ and ‘Flame’ leaves. (**A**) ‘Royalty’. (**B**) ‘Flame’. Error bars indicate the standard error of the mean ± SE of three replicate measurements. Different letters above the bars indicate significantly different values (*P* < 0.05) calculated using one-way analysis of variance (ANOVA) followed by a Duncan’s multiple range test.

**Figure 4:**
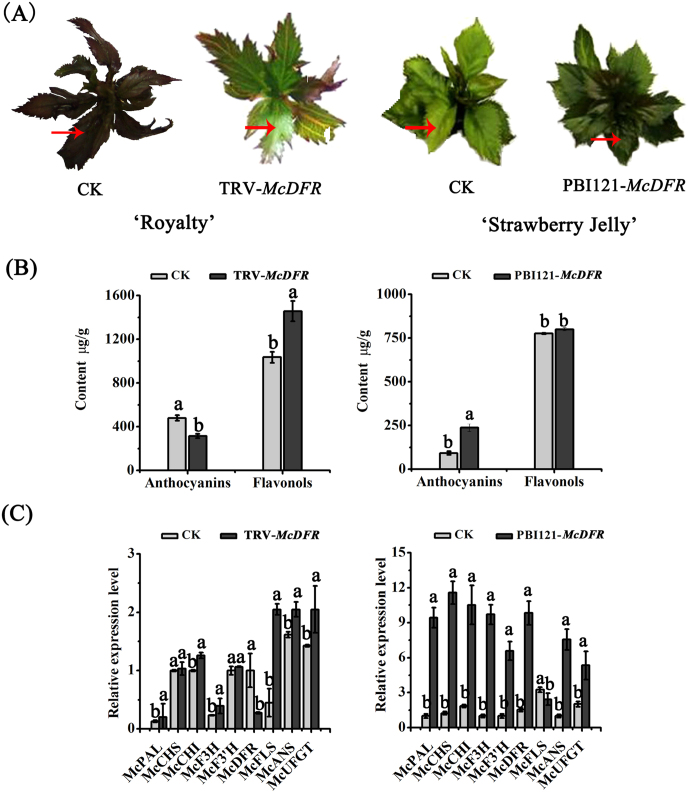
Transient expression of *McDFR* in crabapple. *McDFR* expression was suppressed by VIGS using the vector pTRV2-*McDFR* in ‘Royalty’, or the gene was overexpressed using the vector pBI121-*McDFR* in ‘Strawberry Jelly’. Crabapple leaves injected with the empty TRV and pBI121 vectors and infiltration buffer were used as controls. (**A**) Phenotype of *McDFR* silenced or *McDFR* overexpressing ‘Royalty’ and ‘Strawberry Jelly’ leaves. (**B**) Anthocyanin and flavonol contents at the infiltration sites on crabapple leaves in μg/g fresh weight (FW). (**C**) Relative transcript expression levels in crabapple leaves around the infiltration sites were determined using qRT-PCR. Error bars indicate the standard error of the mean ± SE of three replicate measurements. Different letters above the bars indicate significantly different values (*P* < 0.05) calculated using one-way analysis of variance (ANOVA) followed by a Duncan’s multiple range test.

**Figure 5:**
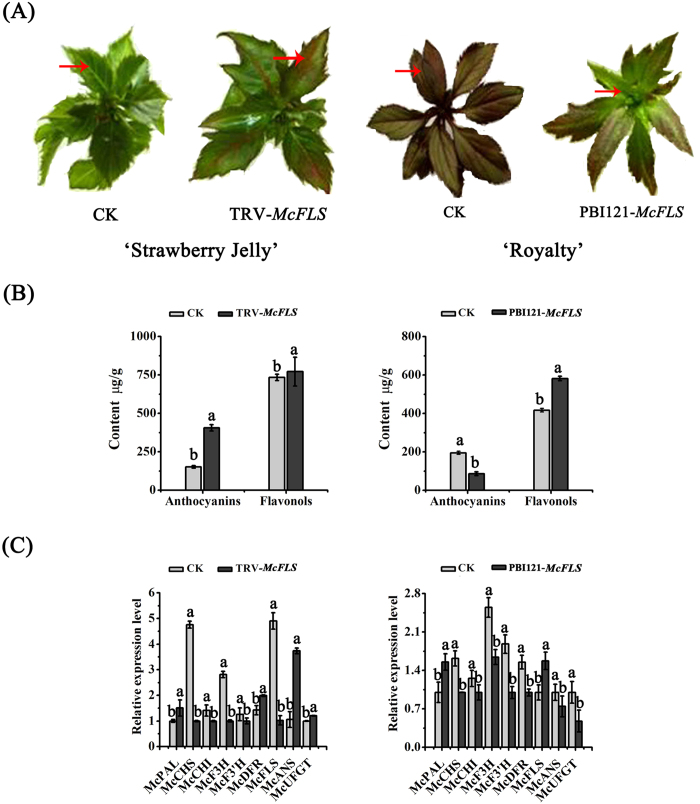
Transient expression of *McFLS* in crabapple. *McFLS* expression was suppressed by VIGS using the vector pTRV2-*McFLS* in ‘Royalty’, or the gene was overexpressed using the vector pBI121-*McFLS* in ‘Strawberry Jelly’. Crabapple leaves injected with the empty TRV and pBI121 vectors and infiltration buffer were used as controls. (**A**) Phenotype of *McFLS* silenced or *McFLS* overexpressing ‘Strawberry Jelly’ and ‘Royalty’ leaves. (**B**) Anthocyanin and flavonol contents at infiltration sites of crabapple leaves in μg/g fresh weight (FW). (**C**) Relative transcript expression levels in crabapple leaves around the infiltration sites were determined using qRT-PCR. Error bars indicate the standard error of the mean ± SE of three replicate measurements. Different letters above the bars indicate significantly different values (*P* < 0.05) calculated using one-way analysis of variance (ANOVA) followed by a Duncan’s multiple range test.

**Figure 6:**
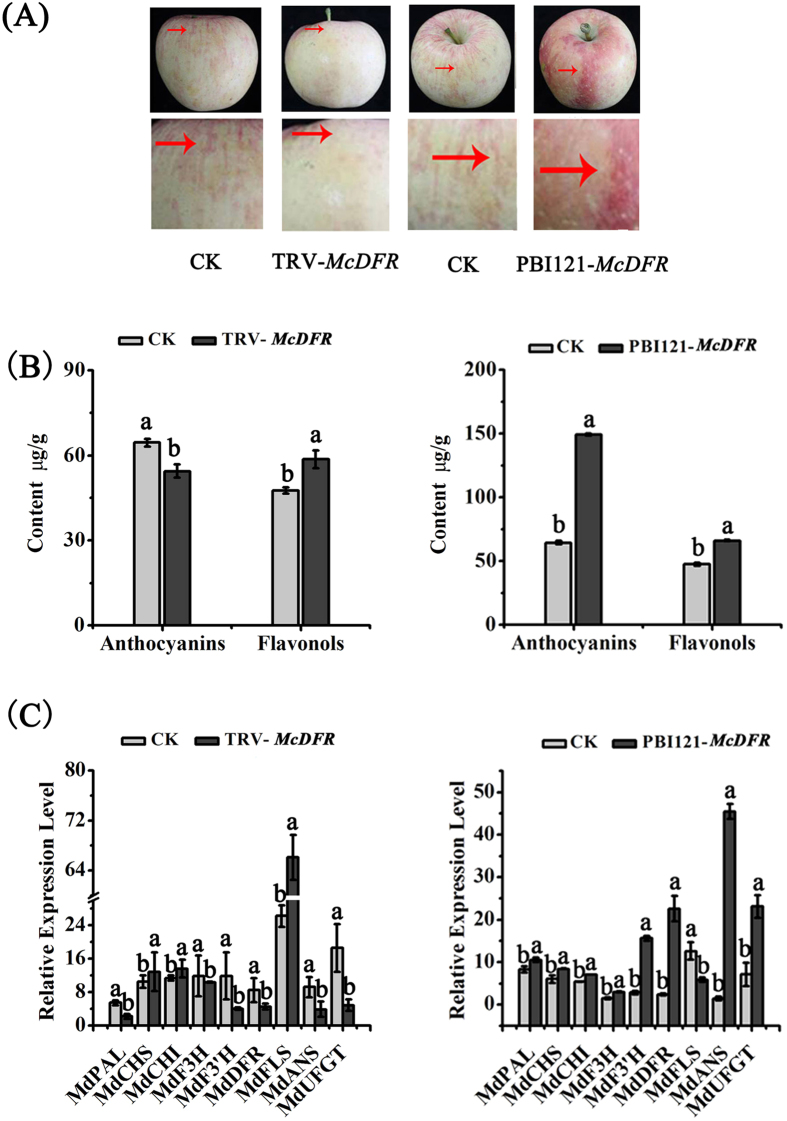
Transient expression of *McDFR* in apple fruit. *McDFR* expression was suppressed in apple fruit using the vector pTRV2-*McDFR*, or the gene was overexpressed using the vector pBI121-*McDFR*. Apple fruit injected with the empty TRV and pBI121 vectors and infiltration buffer were used as controls. (**A**) Phenotype of *McDFR* silenced or overexpressing *McDFR* apple peels. (**B**) Anthocyanin and flavonol contents at the infiltration sites of apple peels in μg/g fresh weight (FW). (**C**) Relative expression levels around the infiltration sites were determined using qRT-PCR. Error bars indicate the standard error of the mean ± SE of three replicate measurements. Different letters above the bars indicate significantly different values (*P* < 0.05) calculated using one-way analysis of variance (ANOVA) followed by a Duncan’s multiple range test.

**Figure 7:**
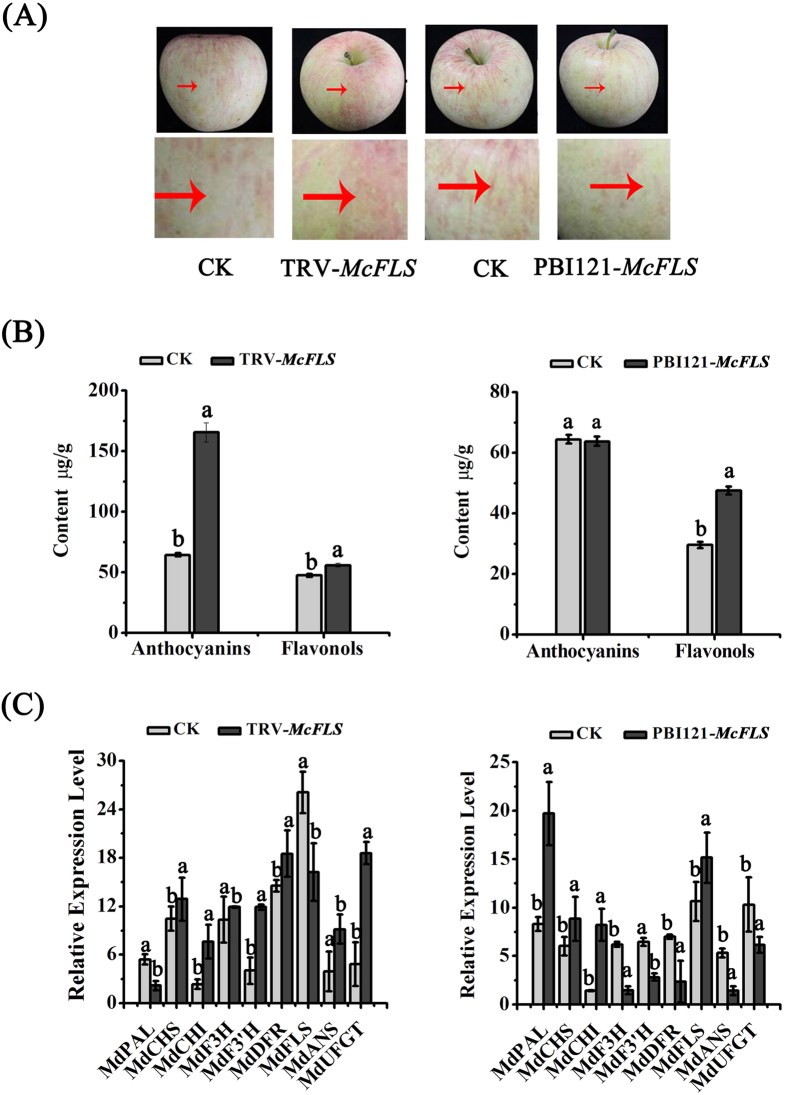
Transient expression of *McFLS* in apple fruit. *McFLS* expression was suppressed in apple fruit using the vector pTRV2- *McFLS*, or overexpressed using the vector pBI121- *McFLS*. Apple fruit injected with the empty TRV and pBI121 vectors and infiltration buffer were used as controls. (**A**) Phenotype of *McFLS* silenced or *McFLS* overexpressing apple peels. (**B**) Anthocyanin and flavonol contents at the infiltration sites of apple peels in μg/g fresh weight (FW). (**C**) Relative expression levels were determined using qRT-PCR around the infiltration sites. Error bars indicate the standard error of the mean ± SE of three replicate measurements. Different letters above the bars indicate significantly different values (*P* < 0.05) calculated using one-way analysis of variance (ANOVA) followed by a Duncan’s multiple range test.

Leaves of ‘Royalty’ and ‘Flame’ were collected at five different developmental stages (Fig. 1A,B) for gene expression analyses and anthocyanin and flavonol quantification. Wild-type ‘Royalty’ and ‘Strawberry’ seedlings were grown in a greenhouse, as above. Apple (*Malus* × *domestica* ‘Fuji’) fruits were used for analysis of *McDFR* and *McFLS* expression. All samples were frozen in liquid nitrogen upon collection, and stored at −80 °C until further use.

### Construction of VIGS vectors and *Agrobacterium*-mediated infiltration of crabapple

The coding sequences of *McDFR* (GenBank: FJ817487) and *McFLS* (GenBank: KF495602) have previously been deposited in the NCBI (National Center for Biotechnology Information) database. Fragments for the pTRV2-*McDFR* (540 bp) and pTRV2-*McFLS* (490 bp) constructs were amplified by PCR, using gene-specific primers, from a cDNA library derived from *Malus* crabapple leaves (cv. ‘Royalty’) using Taq DNA polymerase (TAKARA BIOTECH) according to the manufacturer’s instructions. The PCR primers used are shown in Table S1. Virus-induced gene silencing vectors carrying the target gene fragments, as well as pTRV1 and pTRV2^47^,^48^,^49^, were transformed into *Agrobacterium tumefaciens* strain GV3101 competent cells using a freeze-thaw method^55^ and selected on kanamycin- rifampicin-containing (50 mg/L) LB (Luria Bertani media) plates. Positive clones were verified by restriction enzyme digestion and by sequencing the vector-insert junctions. The harvested bacterial cells were then resuspended to an OD_600_ of 0.5 in infiltration medium (10 mM 2-morpholinoethanesulfonic acid [MES], 200 mM acetosyringone, and 10 mM MgCl_2_) and incubated at room temperature for 3 h. Before infiltration, bacteria carrying pTRV1 and pTRV2 were mixed in a 1: 1 volume ratio.

For vacuum infiltration, whole plants were submerged in the *Agrobacterium* suspension and subjected to a vacuum (−25 kPa). When the rate of air bubbles being released from the plants started to decrease, the vacuum was released quickly to allow bacteria to enter the plant tissues. The vacuum treatment time varied from 30 s up to 3 min, depending on the vacuum source used. After vacuum infiltration, plants were rinsed with sterile water, and cultured on Murashige and Skoog medium. Fifteen plants from each cultivar were treated, and ‘Royalty’ was used for silencing of *McDFR* expression, while ‘Strawberry Jelly’ was used for silencing of *McFLS* expression. All experiments were repeated three times.

### Overexpression of *McDFR* and *McFLS* in crabapple leaves and fruits

The full length *McDFR* and *McFLS* open reading frames (ORFs) were cloned from the cDNA library described above and inserted into the pBI121 vector^53^ using the *Xba*I and *Sac*I sites. Primers used for these constructs are shown in Table S1. Transient expression in *Malus* crabapple leaves was performed using the ‘Strawberry’ Jelly cultivar and *Agrobacterium*-mediated transformation, as described above. *Agrobacterium* cells containing the different constructs were harvested and resuspended in infiltration buffer (10 mM MES, 0.2 mM acetosyringone, and 10 mM MgCl_2_) to a final concentration of OD_600_ = 0.5. Vacuum infiltration was performed as described above, and infiltration with an empty vector was used as a negative control. Seven days after infiltration, the infected leaves and fruit were collected to observe phenotypic features and to evaluate differences in expression.

### RNA extraction

To analyze the effects of VIGS and overexpression on target genes expression, tissue samples from areas showing the silencing and enhancing phenotypes were collected. For controls, corresponding samples were collected from tissues infected by *Agrobacterium* carrying vectors with no host gene fragment insert, or from non-infected plants. Samples from three independent biological replicates were analyzed. Total RNA was extracted from crabapple leaves using the RNA plant plus Reagent (TIANGEN BIOTECH) according to the manufacturer’s instructions. DNase (TIANGEN BIOTECH) treatment was performed to remove any genomic DNA according to the manufacturer’s instructions. First-strand cDNA was synthesized from total RNA using the Reverse Transcriptase M-MLV (RNase H^−^) kit (TaKaRa).

### Quantitative RT-PCR analysis

qRT-PCR was performed using the SYBR® Premix Ex TaqTM II (Perfect Real Time) (TaKaRa, Ohtsu, Japan) and the CFX96TM Real Time System (Bio-Rad, USA). The PCR amplification conditions were as previously described^56^, and transcript levels were determined by relative quantification using the *Malus* 18S ribosomal RNA gene (DQ341382) as the internal control and the 2^ ^(−∆∆CT)^ analysis method was applied. Specific primers (Table S1) for semi-quantitative RT-PCR and qRT-PCR analysis were designed using the primer 5 software^57^.

### HPLC analysis

Crabapple leaf samples (approximately 0.8–1.0 g fresh weight) were subjected to extraction with 10 mL extraction solution (methanol: water: formic acid: trifluoroacetic acid= 70: 27: 2: 1)^58^ at 4 °C in the dark for 72 h, shaking every 6 h. The supernatant was isolated by filtration through filter paper and a further filtration through a 0.22 μm Millipore^TM^ filter (Billerica, MA, USA). For the HPLC analysis, trifluoroacetic acid: formic acid: water (0.1: 2: 97.9) was used as mobile phase A and trifluoroacetic acid: formic acid: acetonitrile: water (0.1: 2: 48: 49.9) was used as mobile phase B. The gradients used were as follows: 0 min, 30% B; 10 min, 40% B; 50 min, 55% B; 70 min, 60% B; 30 min, 80% B. Detection was performed at 520 nm for anthocyanin and 350 nm for flavonol^58^, respectively. All samples were analyzed in three biological triplicates (extracted from three different batches of leaves).

In this study, we employed HPLC-ESI (±)-MS2 analysis to identify the kinds of compounds by standards and comparing their spectroscopic data to literature^59^,^60^. Cyanidin-3-O-glucoside, quercetin-3-O-glucoside, avicularin, phloridzin, quercetin (Sigma-Aldrich, Germany), Procyanidin B2 (Sigma-Aldrich, UK) was used as standards.

## Additional Information

**How to cite this article**: Tian, J. *et al.* The Balance of Expression of Dihydroflavonol 4-reductase and Flavonol Synthase Regulates Flavonoid Biosynthesis and Red Foliage Coloration in Crabapples. *Sci. Rep.* **5**, 12228; doi: 10.1038/srep12228 (2015).

## Supplementary Material

Supplementary Information

## Figures and Tables

**Table 1:** Correlation coefficients between the levels of expression of two biosynthetic genes and flavonoid content.

	Genes		Flavonoids	
	McDFR	McFLS	Anthocyanins	Flavonols
McDFR	1.00	−0.23	0.55^**^	−0.52^**^
McFLS	−0.23	1.00	−0.49^**^	0.27
Anthocyanins	0.55^**^	−0.49^**^	1.00	−0.73^**^
Flavonols	−0.52^**^	0.27	−0.73^**^	1.00

^*^Correlation coefficient was significant at the *P* ≤ 0.05 level. **Correlation coefficient was significant at the *P* ≤ 0.01 level.

## References

[b1] Misra P. *et al.* Modulation of transcriptome and metabolome of tobacco by *Arabidopsis* transcription factor, *AtMYB12*, leads to insect resistance. Plant Physiol. 152, 2258–2268 (2010). 20190095 10.1104/pp.109.150979 PMC2850017

[b2] Page M., Sultana N., Paszkiewicz K., Florance H. & Smirnoff N. The influence of ascorbate on anthocyanin accumulation during high light acclimation in *Arabidopsis thaliana*: further evidence for redox control of anthocyanin synthesis. Plant Cell Environ. 35, 388–404 (2012). 21631536 10.1111/j.1365-3040.2011.02369.x

[b3] Wojdyło A., Oszmianski J. & Czemerys R. Antioxidant activity and phenolic compounds in 32 selected herbs. Food Chem. 105, 940–949 (2007).

[b4] Winkel-Shirley B. Flavonoid biosynthesis. A colorful model for genetics, biochemistry, cell biology, and biotechnology. Plant Physiol. 126, 485–493 (2001). 11402179 10.1104/pp.126.2.485 PMC1540115

[b5] Martens S., Preuss A. & Matern U. Multifunctional flavonoid dioxygenases: flavonol and anthocyanin biosynthesis in *Arabidopsis thaliana* L. Phytochemistry 71, 1040–1049 (2010). 20457455 10.1016/j.phytochem.2010.04.016

[b6] Kobayashi H., Oikawa Y., Koiwa H. & Yamamura S. Flower-specific gene expression directed by the promoter of a chalcone synthase gene from *Gentiana triflora* in *Petunia hybrida*. Plant Sci. 131, 173–180 (1998).

[b7] Tanaka Y. *et al.* Molecular and biochemical characterization of three anthocyanin synthetic enzymes from *Gentiana triflora*. Plant Cell Physiol. 37, 711–716 (2006). 8819318 10.1093/oxfordjournals.pcp.a029004

[b8] Fujiwara H. *et al.* cDNA cloning, gene expression and subcellular localization of anthocyanin 5-aromatic acyltransferase from *Gentiana triflora*. Plant J. 16, 421–431 (2001). 9881162 10.1046/j.1365-313x.1998.00312.x

[b9] Jaakola L. *et al.* Expression of genes involved in anthocyanin biosynthesis in relation to anthocyanin, proanthocyanidin, and flavonol levels during bilberry fruit development. Plant Physiol. 130, 729–739 (2002). 12376640 10.1104/pp.006957 PMC166602

[b10] Park J. S. *et al.* EST analysis of genes involved in secondary metabolism in *Camellia sinensis* (tea), using suppression subtractive hybridization. Plant Sci. 166, 953–961 (2004).

[b11] Nakatsuka T., Nishihara M., Mishiba K. & Yamamura S. Temporal expression of flavonoid biosynthesis-related genes regulates flower pigmentation in *gentian* plants. Plant Sci. 168, 1309–1318 (2005).

[b12] Fukuchi-Mizutani M. *et al.* Biochemical and molecular characterization of a novel UDP-glucose: anthocyanin 3-O-glucosyltransferase, a key enzyme for blue anthocyanin biosynthesis, from gentian. Plant Physiol. 132, 1652–1663 (2003). 12857844 10.1104/pp.102.018242 PMC167102

[b13] Moriguchi T., Kita M., Tomono Y., Endo-Inagaki T. & Omura M. Gene expression in flavonoid biosynthesis: correlation with flavonoid accumulation in developing citrus fruit. Plant Physiol. 111, 66–74 (2008).

[b14] Han Y. P., Vimolmangkang S., Soria-Guerra R. E. & Korban S. S. Introduction of apple *ANR* genes into tobacco inhibits expression of both *CHI* and *DFR* genes in flowers, leading to loss of anthocyanin. J. Exp. Bot. 63, 2437–2447 (2012). 22238451 10.1093/jxb/err415 PMC3346214

[b15] Dare A. P. *et al.* Phenotypic changes associated with RNA interference silencing of chalcone synthase in apple (*Malus* × *domestica*). Plant J. 74, 398–410 (2013). 23398045 10.1111/tpj.12140

[b16] Holton T. A. & Cornish E. C. Genetics and biochemistry of anthocyanin biosynthesis. Plant Cell. 7, 1071–1083 (1995). 12242398 10.1105/tpc.7.7.1071 PMC160913

[b17] Grotewold E., Athma P. & Peterson T. Alternatively spliced products of the maize *P* gene encode proteins with homology to the DNA-binding domain of myb-like transcription factors. Proc. Natl. Acad. Sci. 88, 4587–4591 (1991). 2052542 10.1073/pnas.88.11.4587 PMC51710

[b18] Lepiniec L. *et al.* Genetics and biochemistry of seed flavonoids. Plant Biol. 57, 405–430 (2006). 10.1146/annurev.arplant.57.032905.105252 16669768

[b19] Aharoni A. *et al.* The strawberry FaMYB1 transcription factor suppresses anthocyanin and flavonol accumulation in transgenic tobacco. Plant J. 28, 319–332 (2001). 11722774 10.1046/j.1365-313x.2001.01154.x

[b20] Gou J. Y., Felippes F. F., Liu C. J., Weigel D. & Wang J. W. Negative regulation of anthocyanin biosynthesis in *Arabidopsis* by a miR156-targeted SPL transcription factor. Plant Cell. 23, 1512–1522 (2011). 21487097 10.1105/tpc.111.084525 PMC3101539

[b21] Stracke R. *et al.* Differential regulation of closely related R2R3-MYB transcription factors controls flavonol accumulation in different parts of the *Arabidopsis thaliana* seedling. Plant J. 50, 660–677 (2007). 17419845 10.1111/j.1365-313X.2007.03078.x PMC1976380

[b22] Matsui K., Umemura Y. & Ohme-Takagi M. AtMYBL2, a protein with a single MYB domain, acts as a negative regulator of anthocyanin biosynthesis in *Arabidopsis*. Plant J. 55, 954–967 (2008). 18532977 10.1111/j.1365-313X.2008.03565.x

[b23] Zhu Y., Evans K. M. & Peace C. Utility testing of an apple skin color MdMYB1 marker in two progenies. Mol. Breeding 27, 525–532 (2011).

[b24] Espley R. V. *et al.* Red colouration in apple fruit is due to the activity of the MYB transcription factor, MdMYB10. Plant J. 49, 414–427 (2007). 17181777 10.1111/j.1365-313X.2006.02964.x PMC1865000

[b25] Ban Y. *et al.* Isolation and functional analysis of a MYB transcription factor gene that is a key regulator for the development of red coloration in apple skin. Plant Cell Physiol. 48, 958–970 (2007). 17526919 10.1093/pcp/pcm066

[b26] Shirley B. W., Hanley S. & Goodman H. M. Effects of ionizing radiation on a plant genome: Analysis of two *Arabidopsis transparent* testa mutations. Plant Cell. 4, 333–347 (1992). 1354004 10.1105/tpc.4.3.333 PMC160133

[b27] Shirley B. W. *et al.* Analysis of *Arabidopsis* mutants deficient in flavonoid biosynthesis. Plant J. 8, 659–671 (1995). 8528278 10.1046/j.1365-313x.1995.08050659.x

[b28] Yuan Y., Ma X. H., Shi Y. & Tang D. Q. Isolation and expression analysis of six putative structural genes involved in anthocyanin biosynthesis in *Tulipa fosteriana*. Sci. Hor. 153, 93–102 (2013).

[b29] Guo F. D., Xia H. Y. M. & Wang X. J. Cloning of dihydroflavonol 4-reductase gene (*DFR*) from peanut (*Arachis hypogaea* L.) and its expression analysis. J Agri. Biotechnol. 19, 816–822 (2011).

[b30] Markham K. R. & Hammett K. R. W. The basis of yellow coloration in *Lathyrus-Aphaca* flowers. Phytochemistry 37, 163–165 (1994).

[b31] Aida R., Yoshida K., Kondo T., Kishimoto S. & Shibata M. Copigmentation gives bluer flowers on transgenic *torenia* plants with the antisense dihydroflavonol 4-reductase gene. Plant Sci. 160, 49–56 (2000). 11164576 10.1016/s0168-9452(00)00364-2

[b32] Winkel B. S. J. Metabolic channeling in plants. Annu. Review Plant Biol. 55, 85–107 (2004). 10.1146/annurev.arplant.55.031903.141714 15725058

[b33] Holton T. A., Brugliera F. & Tanaka Y. Cloning and expression of flavonol synthase from *Petunia hybrida*. Plant J. 4, 1003–1010 (1993). 7904213 10.1046/j.1365-313x.1993.04061003.x

[b34] Moriguchia T. *et al.* Flavonol synthase gene expression during citrus fruit development. Physilo. Plantarum. 114, 251–258 (2002). 10.1034/j.1399-3054.2002.1140211.x 11903972

[b35] Tsuda S. *et al.* Molecular breeding of flower color of *Torenia* hybrida. Curr. Plant Sci. Biot. 36, 613–616 (1999).

[b36] Nielsen K. *et al.* Antisense flavonol synthase alters copigmentation and flower color in *lisianthus*. Mol. Breed. 9, 217–229 (2002).

[b37] Zhou X. W. *et al.* Functional analyses of a flavonol synthase-like gene from *Camellia nitidissima* reveal its roles in flavonoid metabolism during floral pigmentation. J. Bio. Sci. 38, 593–604 (2013). 10.1007/s12038-013-9339-2 23938391

[b38] Tian J., Shen H. X., Zhang J., Song T. T. & Yao Y. C. Characteristics of chalcone synthase promoters from different leaf-color *Malus* crabapple cultivars. Sci. Hort. 129, 449–458 (2011).

[b39] Liu Y., Schiff M. & Dinesh-Kumar S. P. Virus-induced gene silencing in tomato. Plant J. 31, 777–786 (2002). 12220268 10.1046/j.1365-313x.2002.01394.x

[b40] Rosati C., Cadic A., Duron M., Renou J. P. & Simoneau P. Molecular cloning and expression analysis of dihydroflavonol 4-reductase gene in flower organs of *Forsythia x intermedia*. Plant Mol. Biol. 35, 303–311 (1997). 9349254 10.1023/a:1005881032409

[b41] Himi E. & Noda K. Isolation and location of three homologous dihydroflavonol 4-reductase (*DFR*) genes of wheat and their tissue-dependent expression. J. Exp. Bot. 55, 365–375 (2004). 14718498 10.1093/jxb/erh046

[b42] Zhang P. *et al.* Molecular cloning of dihydroflavonol 4-reductase gene from grape berry and preparation of an anti-DFR polyclonal antibody. Vitis. 47, 141–145 (2008).

[b43] Kunu W., Thanonkeo S. & Thanonkeo P. Cloning and expression analysis of dihydroxyflavonol 4-reductase (*DFR*) in *Ascocenda* spp. *Afr*. J. Biotechnol. 11, 12702–12709 (2012).

[b44] Pandey A., Misra P., Bhambhani S., Bhatia C. & Trivedi P. K. Expression of *Arabidopsis* MYB transcription factor, *AtMYB111*, in tobacco requires light to modulate flavonol content. Sci. Rep. 4, 5018; 10.1038/srep05018 (2014). 24846090 PMC4028898

[b45] Muir S. R. *et al.* Overexpression of petunia chalcone isomerase in tomato results in fruit containing increased levels of flavonols. Nature Biotechnol. 19, 470–474 (2001). 11329019 10.1038/88150

[b46] Dare A. P. *et al.* Phenotypic changes associated with RNA interference silencing of chalcone synthase in apple (*Malus domestica*). Plant J. 74, 398–410 (2013). 23398045 10.1111/tpj.12140

[b47] Tian J. *et al.* TRV-GFP: a modified tobacco rattle virus vector for efficient and visualizable analysis of gene function. J. Exp. Bot. 65, 311–322 (2013). 24218330 10.1093/jxb/ert381 PMC3883300

[b48] Tian J., Cheng L., Han Z. Y. & Yao Y. C. Tobacco rattle virus mediated gene silencing in strawberry plants. Plant Cell Tiss. Organ Cult. 120, 1131–1138 (2015).

[b49] Tian J. *et al.* McMYB10 regulates coloration via activating *McF3′H* and later structural genes in ever-red leaf crabapple. Plant Biotechnol. J. 10.1111/pbi.12331 (2015). 25641214

[b50] Riechmann J. L. *et al.* Arabidopsis transcription factors: genome-wide co mparative analysis among eukaryotes. Science 290, 2105–2110 (2000). 11118137 10.1126/science.290.5499.2105

[b51] Luo J. *et al.* AtMYB12 regulates caffeoyl quinic acid and flavonol synthesis in tomato: expression in fruit results in very high levels of both types of polyphenol. Plant J. 56, 316–326 (2008). 18643978 10.1111/j.1365-313X.2008.03597.x

[b52] Ravaglia D. *et al.* Transcriptional regulation of flavonoid biosynthesis in nectarine (*Prunus persica*) by a set of R2R3 MYB transcription factors. BMC Plant Biol. 13, 68 (2013). 23617716 10.1186/1471-2229-13-68 PMC3648406

[b53] Jiang R., Tian J., Song T. T., Zhang J. & Yao Y. C. The *Malus* crabapple transcription factor McMYB10 regulates anthocyanin biosynthesis during petal coloration. Sci. Hortic. 166, 42–49 (2014).

[b54] Czemmel S. *et al.* The grapevine R2R3-MYB transcription factor VvMYBF1 regulates flavonol synthesis in developing grape berries. Plant Physiol. 151, 1513–1530 (2009). 19741049 10.1104/pp.109.142059 PMC2773091

[b55] Yan H. X. *et al.* Sprout vacuum-infiltration: a simple and efficient agroinoculation method for virus-induced gene silencingin diverse *solanaceous species*. Plant Cell Rep. 31, 1713–1722 (2012). 22717672 10.1007/s00299-012-1285-1

[b56] Shen H. X. *et al.* Isolation and expression of *McF3H* gene in the leaves of crabapple. Acta. Physiol. Plant 34, 1353–1361 (2012).

[b57] Tai D. Q., Tian J., Zhang J., Song T. T. & Yao Y. C. A *Malus* crabapple chalcone synthase gene, *McCHS*, regulates red petal color and flavonoid biosynthesis. Plos One 10: e110570 (2014). 25357207 10.1371/journal.pone.0110570 PMC4214706

[b58] Revilla E. & Ryan J. M. Analysis of several phenolic compounds with potential antioxidant properties in grape extracts and wines by high-performance liquid chromatography–photodiode array detection without sample preparation. J. Chromatogr. A. 881, 461–469 (2000). 10905728 10.1016/s0021-9673(00)00269-7

[b59] Piero A. R. L., Puglist I. & Petrone G. Gene characterization, analysis of expression and *in vitro* synthesis of dihydroflavonol 4-reductase from *Citrus sinensis* (L.). Osbeck phytochem. 67, 684–695 (2006). 10.1016/j.phytochem.2006.01.025 16524606

[b60] Yildiz M. *et al.* Expression and mapping of anthocyanin biosynthesis genes in carrot. Theor. Appl. Genet. 126, 1689–1702 (2013). 23525633 10.1007/s00122-013-2084-y

