# Supplementary material for: The Balance of Expression of Dihydroflavonol 4-reductase and Flavonol Synthase Regulates Flavonoid Biosynthesis and Red Foliage Coloration in Crabapples
Source: Sci Rep. 2015 Jul 20;5:12228. doi: 10.1038/srep12228 (PMC4507444; doi:10.1038/srep12228)
Supplement: Supplementary Information [file srep12228-s1.doc]

**The Balance of Expression of Dihydroflavonol 4-reductase and Flavonol Synthase Regulates Flavonoid Biosynthesis and Red Foliage Coloration in Crabapples**

Ji Tian^a, b, c^, Zhen-yun Han^a, b, c^, Jie Zhang ^a, b, c^, YuJing Hu ^a, b, c^, Tingting Song ^b, c^, Yuncong Yao ^b, c^*

^a^ Contributed equally to this work.

^b^ Department of Plant Science and Technology, Beijing University of Agriculture, Beijing, China.

^c^ Key Laboratory of New Technology in Agricultural Application of Beijing, Beijing University of Agriculture, Beijing, China.

*Correspondence should be addressed to Yun-cong Yao (Email: yaoyc_20@126.com; Tel.: +86-10-80799000)

**Table S1** Primer sequences used in this study.

| **Accession**  **number** | **ID** | **Sequence (5’-3’)** | **Used for** |
| --- | --- | --- | --- |
|  |  |  |  |
| X06172 | TRV1-F | TTACAGGTTATTTGGGCTAG |  |
|  | TRV1-R | CCGGGTTCAATTCCTTATC |  |
| AF406991 | TRV2-F | TTACGACGAACCAAGGGAGTACTAC |  |
|  | TRV2-R | AGTCACAATTAGCCCTATTTAGATGT |  |
| FJ817487 | pTRV-McDFR-F | CCGCTCGAGGCTCGTGTTCACATCCTC | XhoI, KpnI |
|  | pTRV-McDFR-R | CGGGGTACCCTCAAACCCAATCTCCCT |  |
| KF495602 | pTRV-McFLS-F | CCGCTCGAGTAGGTCAGAGAACGAGCAAC | XhoI, KpnI |
|  | pTRV-McFLS-R | CGGGGTACCAAGCCCTAACCCTAAAGATA |  |
| FJ817487 | PBI121-McDFR-F | GCTCTAGATTCGATTCTTCGGTAAGCACAT | SacI, XbaI |
|  | PBI121-McDFR-R | GCGAGCTCTGCAAACATTAATCACTCTTC |  |
| KF495602 | PBI121-McFLS-F | CCGAGCTCATTTATTCTGGGGCTTATTGT | SacI, XbaI , |
|  | PBI121-McFLS-R | AATCTAGAATGGGAGTGGAGTCTGTGGAGAG |  |
| JQ248934 | McPAL-F | ACCCTGGACAGATTGAGGCAGCT | qRT-PCR |
|  | McPAL-R | GCCTAGCGATCCTGCTTTGGCT |  |
| FJ599763 | McCHS-F | TGACCGTCGAAGTTCGC | qRT-PCR |
|  | McCHS-R | TTTGTCACACATGCGCTGGA |  |
| FJ817485 | McCHI-F | AGGAGTTGTCGGAGTCCGTT | qRT-PCR |
|  | McCHI-R | ACTTTCTCAGAGTATTGCTGGCC |  |
| FJ817486 | McF3H-F | ACGAAGACGAGCGTCCAAAG | qRT-PCR |
|  | McF3H-R | CTCCTCCGATGGCAAAGCAA |  |
| KF481684 | McF3’H-F | CGTTGCTGTCGCTCACGGATGA | qRT-PCR |
|  | McF3’H-R | ATGACGTGTCAGTGCCAGCTGTG |  |
| FJ817487 | McDFR-F | CCGAGTCCGAATCCGTTTGT | qRT-PCR |
|  | McDFR-R | CCTTCTTCTGATTCGTGGGGT |  |
| FJ817488 | McANS-F | CACAGGGGCATGGTGAACAA | qRT-PCR |
|  | McANS-R | TTCACTTGGGGAGCAAAGCC |  |
| KF495603 | McUFGT-F | TGGGCGGACACCAATCA | qRT-PCR |
|  | McUFGT-R | ATGTCTCCACCGCACCA |  |
| KF495602 | McFLS-F | ACGAGCAACCGGGAATCACAACTG | qRT-PCR |
|  | McFLS-R | CCCAGTTGGAGCTGGCCTCAGTA |  |
| DQ341382 | 18S RNA-F | GTCACTACCTCCCCGTGTCA | qRT-PCR |
|  | 18S RNA-R | GAGCCTGAGAAACGGCTACC |  |
